# Supplementary figures and images for: Bacterial communities of indoor surface of stingless bee nests
Source: PLoS One. 2021 Jul 9;16(7):e0252933. doi: 10.1371/journal.pone.0252933 (PMC8270128; doi:10.1371/journal.pone.0252933)

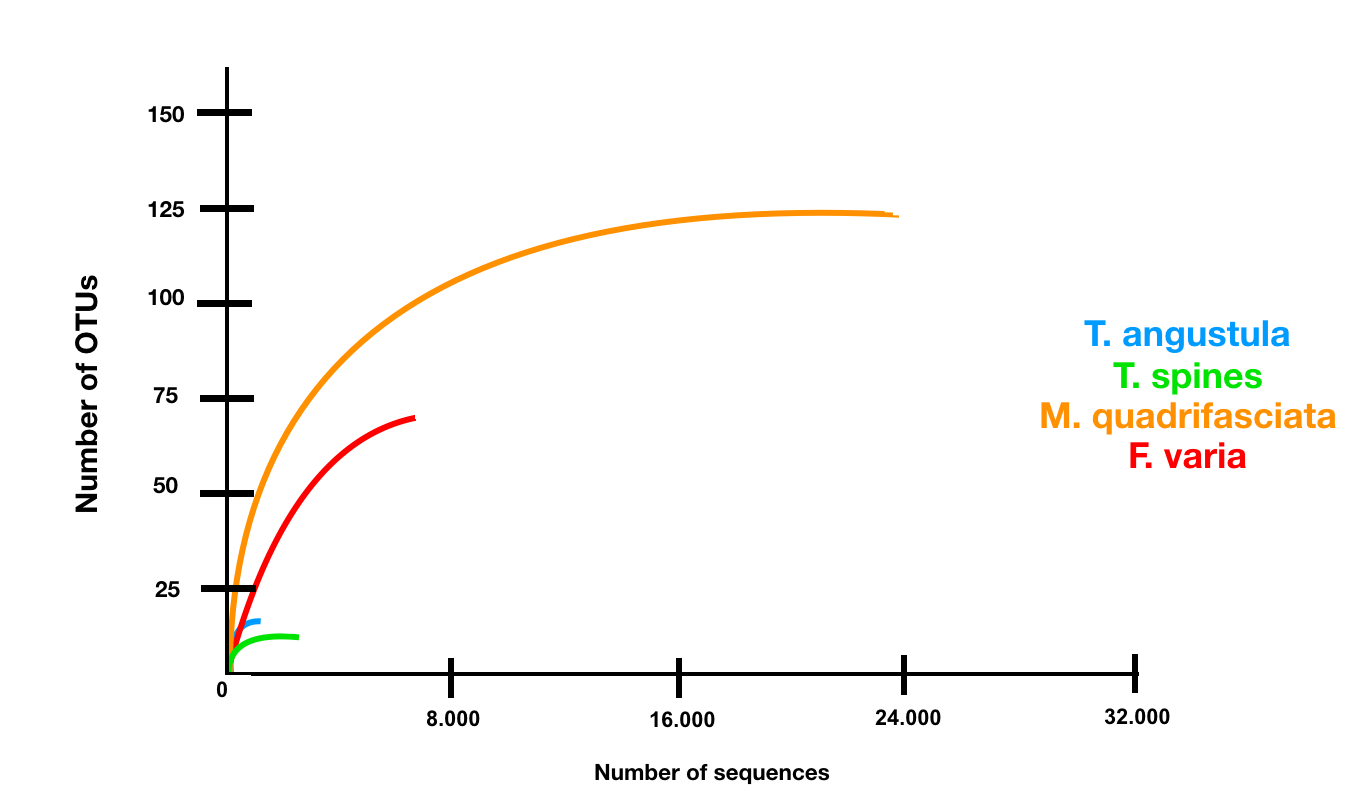

Supplement: S1 Fig — (TIF) [file pone.0252933.s001.tif]
