# Supplementary material for: Bacterial communities of indoor surface of stingless bee nests
Source: PLoS One. 2021 Jul 9;16(7):e0252933. doi: 10.1371/journal.pone.0252933 (PMC8270128; doi:10.1371/journal.pone.0252933)
Supplement: Attachment — Submitted filename: Response to Reviewers.docx [file pone.0252933.s010.docx]

Response to Reviewers and Editor

Editor: Both reviewers found substantial shortcomings in the description of the methods. Please provide more detail and bear in mind that all methods and data analysis should be able to be followed by fellow researchers. A particularly problematic issue with the manuscript is the statistical analysis. Please make sure to properly describe and perform the necessary statistical analysis. Finally, we recommend that authors select repositories appropriate to their field (e.g., GenBank for sequences)

R: The statistical analysis was described as well as information about sequencing. The sequences were deposited at the NCBI GenBank

Reviewer #1: It is an interesting finding in this manuscript, and I think it can be publish in Plos One with minor revision.

1. DNA sequencing procedure should be described in detail.

R: Done. Thanks!

2. Why the sampling was performed two days after a heavy rain day？

R:It is an environmental information that can be useful for understanding the issue since two of the species of stingless bees use clay / mud (more abundant in rainy seasons) for the construction of the nest.

Reviewer #2: The submitted paper entitled "Bacterial communities of indoor surface of stingless bees nests" is a picture of the microbial components of stingless bees nests performed by high throughput sequencing. It is an interesting research with outputs that can improve the general knowledge of the microbial communities and distribution in the environment but not enough original. The reading is clear, the main question research and finding are well described but simple. Methods are scarcely described (i.e number of total samples) and statistics is missing. In my opinion the manuscript is not appropriate for publication in this Journal.

R: The question of samples was made clearer in the text and the statistics were improved. Thanks for the suggestions.
